# Supplementary material for: HLA-G gene editing in tumor cell lines as a novel alternative in cancer immunotherapy
Source: Sci Rep. 2021 Nov 12;11:22158. doi: 10.1038/s41598-021-01572-0 (PMC8589947; doi:10.1038/s41598-021-01572-0)

HLA-G gene editing in tumor cell lines as a novel alternative in cancer immunotherapy

María Belén Palma, Diana Tronik-Le Roux, Guadalupe Amín, Sheila Castañeda, Alan M. Möbbs, María Agustina Scarafia, Alejandro La Greca, Marina Daouya, Isabelle Poras, Ana María Inda, Lucía N. Moro, Edgardo D. Carosella, Marcela N. García, Santiago G. Miriuka.

Figure S1: HLA-G expression in JEG-3 and RCC7/HLA-G1 cell lines determined by RT-qPCR. HLA-G expression values were compared to cytotrophoblast cells (extravillous cytotrophoblast extracted from term placenta). HFF: Human fibroblast cells (negative control). JEG-3: choriocarcinoma cell line. RCC7/HLA-G1: renal cell carcinoma cell line expressing HLA-G1. Significant differences are shown with * ($p<0.05$).

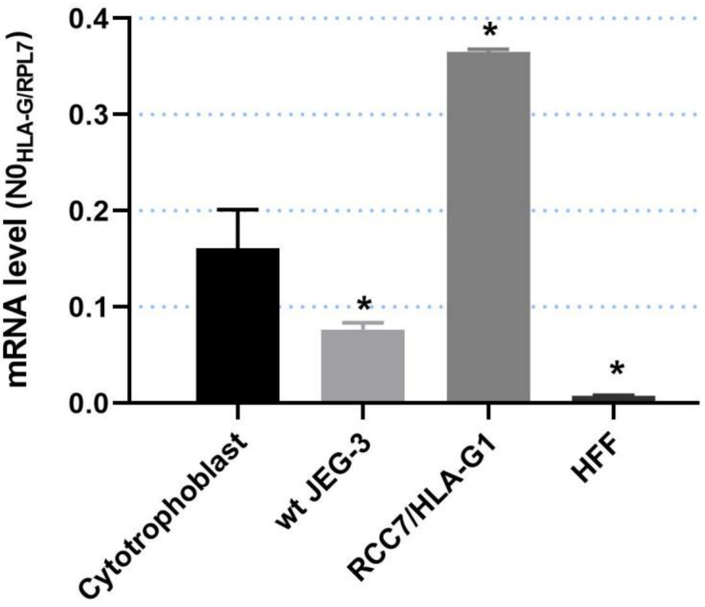

Figure S2: Full-length, unedited Western blot for edited and unedited RCC7/HLA-G1 cells, showed in figure 2A in the manuscript. On the left, it shows AA4, AA8, EE2 and B10 RCC7/HLA-G1 clonal cell lines edited with 2A-sgRNA. On the right, it shows the unedited RCC7/HLA-G1 cell line.

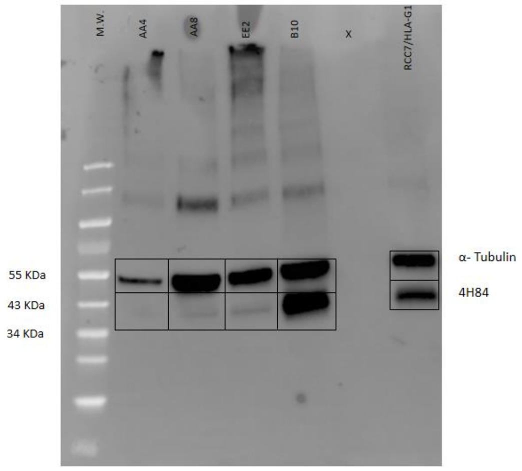

Figure S3: Full-length, unedited Western blot for edited and unedited JEG-3 cells, showed in figure 3A and 4A in the manuscript. It shows the JEG-3 cells edited with each sgRNA (1A, 1B, 2A and 2B) and with 4 sgRNAs simultaneously (MIX-sgRNAs). On the right, it shows the unedited JEG-3 cell line.

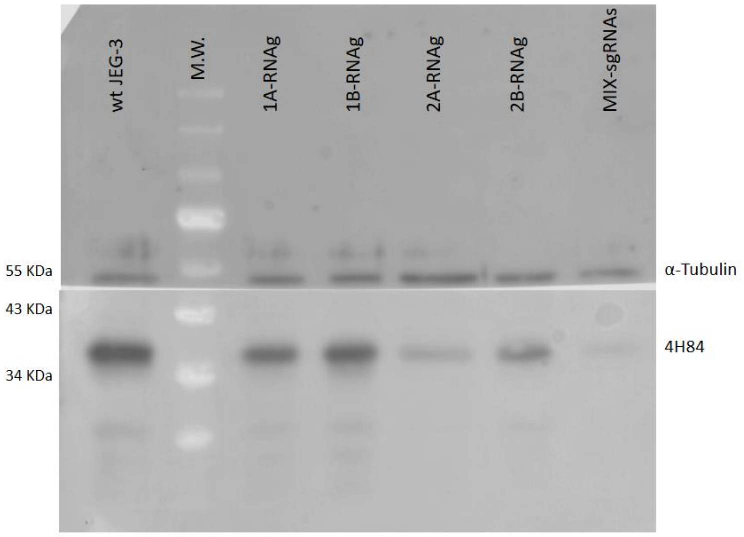

Figure S4: Full-length, unedited agarose gel from RT-PCRs, showed in figure 2B in the manuscript. Each gel shows the results using the following pairs of primers: 257F/526R, Ex1-F/257R, and Ex2-F/526R. On the left, it shows RCC7 wild type and RCC7/HLA-G1 cell line. Then, AA4, AA8, EE2 and B10 RCC7/HLA-G1 clonal cell lines edited with 2A-sgRNA. 1Kb ladder was used and the molecular weight of each band is specified on the left.

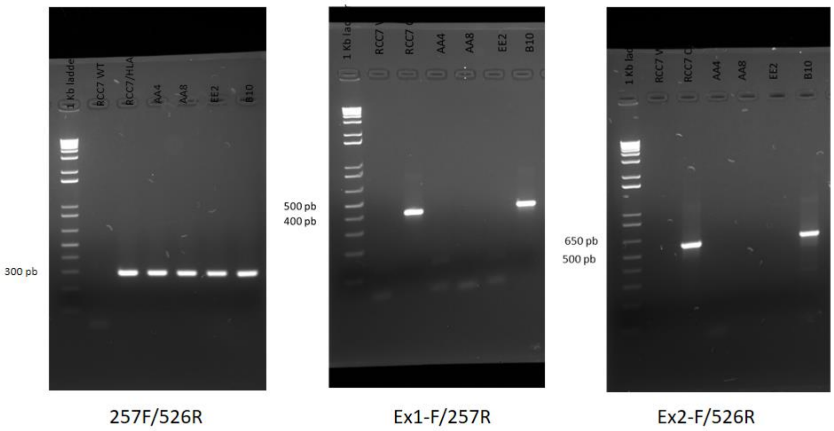

Figure S5: Full-length, unedited agarose gel from PCR, showed in figure 2D in the manuscript. The genomic DNA amplification achieved with CRR1F/257R primers is showed. Left to right, RCC7 wild type and RCC7/HLA-G1 cell line. Then, six RCC7/HLA-G1 clonal cell lines edited with 2A-sgRNA. In the manuscript, the agarose gel was cropped to show only three clonal cell lines, AA4, AA8, and EE2. 1Kb ladder was used and the molecular weight of each band is specified on the left.

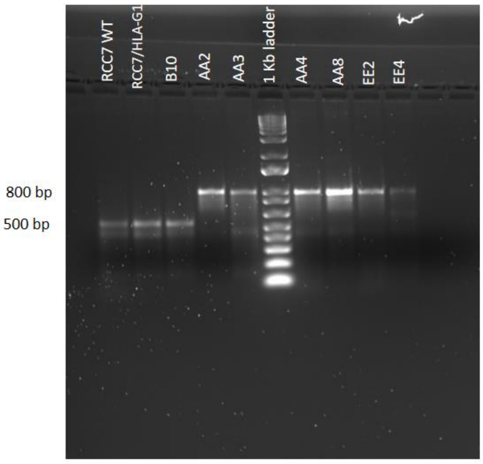

Supplement: Supplementary file 1 — Supplementary Information. [file 41598_2021_1572_MOESM1_ESM.pdf]
